# Magnetophotoselection in the Investigation of Excitonically Coupled Chromophores: The Case of the Water-Soluble Chlorophyll Protein

**DOI:** 10.3390/molecules27123654

**Published:** 2022-06-07

**Authors:** Susanna Ciuti, Alessandro Agostini, Antonio Barbon, Marco Bortolus, Harald Paulsen, Marilena Di Valentin, Donatella Carbonera

**Affiliations:** 1 Department of Chemical Sciences, University of Padova, Via Marzolo 1, 35131 Padova, Italy; susanna.ciuti@phd.unipd.it (S.C.); alessandro.agostini@umbr.cas.cz (A.A.); antonio.barbon@unipd.it (A.B.); marco.bortolus@unipd.it (M.B.); 2 Biology Centre, Czech Academy of Sciences, Institute of Plant Molecular Biology, Branišovská 1160/31, 370 05 České Budějovice, Czech Republic; 3 Institute of Molecular Physiology, Johannes Gutenberg-University of Mainz, Johann-Joachim Becher-Weg 7, 55128 Mainz, Germany; paulsen@uni-mainz.de

**Keywords:** magnetophotoselection, triplet state, chlorophyll-binding protein, excitonic interaction, TR-EPR

## Abstract

A magnetophotoselection (MPS) investigation of the photoexcited triplet state of chlorophyll *a* both in a frozen organic solvent and in a protein environment, provided by the water-soluble chlorophyll protein (WSCP) of *Lepidium virginicum*, is reported. The MPS experiment combines the photoselection achieved by exciting with linearly polarized light with the magnetic selection of electron paramagnetic resonance (EPR) spectroscopy, allowing the determination of the relative orientation of the optical transition dipole moment and the zero-field splitting tensor axes in both environments. We demonstrate the robustness of the proposed methodology for a quantitative description of the excitonic interactions among pigments. The orientation of the optical transition dipole moments determined by the EPR analysis in WSCP, identified as an appropriate model system, are in excellent agreement with those calculated in the point-dipole approximation. In addition, MPS provides information on the electronic properties of the triplet state, localized on a single chlorophyll *a* pigment of the protein cluster, in terms of orientation of the zero-field splitting tensor axes in the molecular frame.

## 1. Introduction

**Figure 1:**
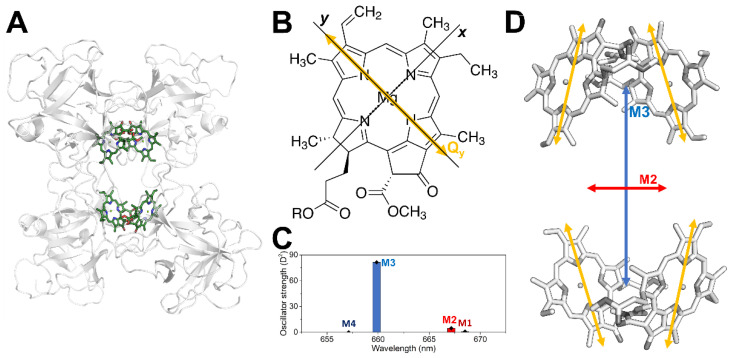
Optical axes of Chl *a* alone and in the excitonic system of WSCP. (**A**) Structure of WSCP (PDB ID: 2DRE [31]) with the four Chl *a* highlighted (protein backbone in light grey ribbon; Chl *a* in dark green with the phytyl chains omitted for clarity). (**B**) The Chl *a* structure (R = phytyl chain) is shown with the TDM of the Q_y_ transition (yellow) and the molecular axes (black). (**C**) Calculated stick spectrum of the four excitonic transitions of WSCP (black diamonds and coloured bars) as described in Section 2.4. (**D**) TDM of the two main states of the excitonic system, M2 (red) and M3 (blue), and Q_y_ TDM of the four monomers (yellow), all shown in the zoomed WSCP site structure (Chl *a* molecules in grey).

Light-harvesting multi-chromophoric systems, such as photosynthetic subunits of phototrophic organisms, rely in their functioning on the three-dimensional disposition and site energy tuning of their bound chromophores, so as to create the energetic landscape required for their functioning [1,2]. A major class of chromophores that ended up being ubiquitous in this role is that of tetrapyrroles [3], particularly chlorophyll *a* (Chl *a*) in oxygenic photosynthesis [4,5]. This molecule is characterized by the intense absorption in the red portion of the visible spectrum of its lowest excited state (S_1_), referred as Q_y_ due to the orientation of its transition dipole moment (TDM), close to the molecular *y* axis (see Figure 1B) [6].

The orientation of the Q_y_ TDM of monomeric Chl *a* was the subject of numerous experimental investigations, using linear dichroism of oriented chlorophyll molecules [7,8], fluorescence depolarization measurements [9] and polarization resolved pump-probe spectroscopy [10]. The Q_y_ TDM was also determined in silico, at a density functional [11,12,13,14] configuration interaction [13] and, recently, at coupled cluster [15] levels of theory. The cited investigations agree on an in-plane orientation of the TDM, which is within ±20° from the molecular *y* axis (see Figure 1B). In complex architectures containing Chls, like those of photosynthetic complexes, the efficiency of singlet-singlet energy transfer critically depends on the distance and reciprocal orientation of their Q_y_ TDMs. Moreover, the low-lying excited states in interacting Chls also depend on these factors. Therefore, knowledge of the precise orientation of the TDM within the molecular structure is a prerequisite for accurate calculations of chlorophyll-chlorophyll excitonic interactions and characterization of the energy transfer pathways.

A system exceptionally well-suited to investigate excitonically coupled Chl *a* molecules is the Water Soluble Chlorophyll Protein (WSCP) [16,17], a protein found in plants belonging to the *Brassicaceae* family [18]. In contrast to other Chl-binding proteins, WSCP is not involved in the photosynthetic process, but its actual physiological role is yet to be determined [19]. Due to its cellular localization in *Brassicaceae* organelles, which are involved in plant defence [20,21] and its stress-induced expression [22,23,24,25], a role in herbivore defense [26] or signaling/regulation [27,28] was proposed. This homo-tetrameric protein, due to its tetrahedral symmetry, binds its four chromophores in four identical binding sites [29,30,31], organized into a “dimer of dimers” configuration of two “open-sandwich” [32,33] dimers that are only weakly interacting [16,34] (see Figure 1D). The presence of excitonic interactions causes important effects in the spectroscopic observables [14,16,32,35,36,37,38,39,40,41,42,43], including a blue-shift of the absorption band in the Q_y_ region. Figure 1C shows an illustrative stick spectrum of the four excitonic transitions calculated in the framework of the point-dipole approximation (labelled M1, M2, M3 and M4), assuming that the Q_y_ TDM of each monomeric Chl *a* is aligned with the molecular *y* axes, while in Figure 1D the corresponding TDMs are shown for the two most intense transitions, M3 and M2 [16]. WSCP is an ideal model system for detailed spectroscopic investigations [16,27,29,35,36,37,38,39,40,41,42,43,44,45,46,47], particularly when compared to Chl-binding complexes involved in photosynthesis where tens to hundreds of chlorophylls are bound in finely tuned Chls binding sites along with other chromophores such as carotenoids [1,2,48].

Knowledge of the TDM orientation within the molecular structure can be obtained from magnetophotoselection (MPS) studies of triplet states performed by time-resolved electron paramagnetic resonance (TR-EPR, [49,50,51]); a summary of the key concepts of the techniques are reported in the Supporting Information. Since the conception of MPS by Kottis and Lefebvre [52], this methodology has been applied to various tetrapyrroles [50,53,54,55,56], including chlorophylls [57] in glassy matrix, as well as primary donors in photosynthetic reaction centres [58,59,60,61,62,63,64]. Its great advantage is the possibility to obtain specific information on the orientation of the TDM without the requirement of realizing macroscopic orientations of the chromophores. This is particularly significant for proteins, which can therefore be investigated in biologically relevant conditions. In the TR-EPR experiment coupled to MPS, the triplet state is photoexcited by linearly polarized laser light, with a laser polarization axis alternatively parallel or perpendicular to the magnetic field direction. Selected molecules contribute to the triplet-state EPR spectrum depending on the relative orientation of their TDM and the light polarization vector. The analysis of the TR-EPR spectra, recorded both in the parallel and perpendicular photoexcitation modes for better accuracy, thus allows the determination of the relative orientation of the TDM and the principal axes of the zero-field splitting (ZFS) tensor in terms of two angles ω, φ. Then, if the orientation of the ZFS axes in the molecular structure is known, that of the TDM in the molecular structure can be obtained.

**Figure 2:**
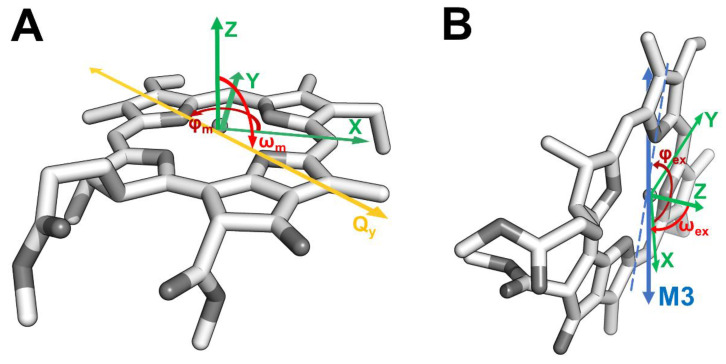
Relative orientation of the optical and magnetic axes of monomeric Chl *a* (assuming for simplicity a Q_y_ TDM oriented along the molecular *y* axis) (**A**) and in the excitonic system of WSCP (**B**) (note that only one of the four Chls *a* of the tetramer is shown for clarity). Chl *a* in grey, hydrocarbon tails are omitted for clarity. (**A**) In the monomer, the ω_m_ and φ_m_ angles (red and dark red, respectively) define the orientation of the Q_y_ TDM (yellow) relative to the ZFS axes system (green). (**B**) In WSCP, the ω_ex_ and φ_ex_ angles (red and dark red, respectively) define the orientation of the M3 TDM (blue) relative to the ZFS axes system (green). The projection of the out-of-plane M3 TDM is reported as a dashed line.

In the case of a monomeric chlorophyll *a* triplet state (^3^Chl *a*), the orientation of the ZFS tensor with respect to the Q_y_ TDM (ω_m_ and φ_m_ in Figure 2A) has been assigned by means of Linear Dichroism Optically Detected Magnetic Resonance (LD-ODMR) [65] as follows: the ZFS Z axis is perpendicular to the molecular plane and the in-plane X, Y axes are rotated by 48° relative to the Q_y_ TDM. Electron Nuclear DOuble Resonance (ENDOR) experiments were also reported to give hyperfine coupling tensors of methine protons compatible with a 45° rotation of the X, Y axes relative to the molecular axes (see Figure 2A) but with an uncertainty of about 10° due to the degree of orientational selection achieved in correspondence of the X and Y canonical transitions [66].

In this work, reconstituted *Lepidium virginicum* WSCP is employed as a case study to illustrate the potential of TR-EPR coupled to MPS for two main objectives: (i) to determine the relative orientation between the TDM and the principal triplet axes in the excitonic system (ω_ex_ and φ_ex_ in Figure 2B), where the triplet state becomes localized on a single molecule after singlet photoexcitation; and (ii) to verify the correctness of the excitonic model through the angular constraints imposed by MPS.

## 2. Materials and Methods

### 2.1. Sample Preparation

Chl *a* was extracted from pea plants (*Pisum sativum*) and purified as described by Booth and Paulsen [67]. Protein overexpression in *E. coli* and subsequent purifications have been performed as previously reported [27]. The purified *Lepidium virginicum* WSCP apoprotein was reconstituted with Chl *a* as previously described [68].

WSCP was concentrated up to a Chl *a* concentration of 700 μg/mL for TR-EPR measurements. Glycerol, previously degassed by several freeze-pump-thaw cycles, was added (60% *v*/*v*) to obtain a transparent matrix immediately before freezing to avoid sample degradation [69].

Chl *a* was dissolved in either MeTHF or an EtOH:MeOH 3:2 solvent mixture for TR-EPR measurements at a concentration ≈75 µM. The samples were degassed by performing several freeze-pump-thaw cycles and were sealed under vacuum in the EPR tube.

TR-EPR experiments were performed using fused silica quartz tubes (3 mm i.d. × 4 mm o.d.). The samples were frozen in liquid nitrogen and quickly transferred to the cold cavity of the EPR spectrometer. The formation of a clear, transparent matrix was checked visually before inserting the samples.

### 2.2. TR-EPR Experiments

TR-EPR experiments were performed on an ELEXSYS E580 spectrometer (from Bruker BioSpin GmbH, Rheinstetten, Germany), equipped with a dielectric cavity (ER 4117-DI5, TE_011_ mode, from Bruker) and operating at X-band (about 9.5 GHz) in continuous-wave mode. The microwave frequency was measured by a frequency counter, HP5342A. An Oxford CF935 cryostat, cooled by a thermostated nitrogen flow, was used to control the temperature; all experiments were conducted at 80 K temperature, at which all samples form rigid glassy matrices. Photo-excitation of the sample was realized with a laser system that emits polarized light and allows for the tuning of the excitation wavelength in the visible region (from Quantel Brilliant, Lannion, France): the system is composed of a Nd:YAG pulsed laser (1064 nm,) equipped with both second and third harmonic and optical parametric oscillator (Rainbow, from Opotek Inc., Carlsbad, CA, USA) modules. The parameters of the laser pulses were: pulse length = 5 ns, E/pulse ≅ 3 mJ, 10 Hz pulse repetition time. MPS experiments were realized by irradiating the sample with polarized laser pulses. Two different polarizations were employed: one with the electric field perpendicular and the other parallel to the static magnetic field of the spectrometer. The rotation of the polarization plane of the light was obtained using a half waveplate; a linear polarizer was inserted in the beam path near the optical window of the cavity for a better control of the polarization, and oriented accordingly to the desired configuration.

TR-EPR experiments were carried out by recording the time evolution of the EPR signal after the laser pulse with a LeCroy 9360 digital oscilloscope triggered by the laser pulse. At each magnetic field position, 200 transient signals were usually averaged before transferring them to the PC controlling the instrument via the XEPR software; 256 points on the magnetic field axis were recorded, with a sweep width of 80.0 mT (in all solvents) or 90.0 mT (in WSCP). The microwave power for TR-EPR experiments was set to be low enough to be in a low-power regime and avoid Torrey oscillations on the time trace: microwave attenuation: 28 dB in solvent, 35 dB in WSCP. The time vs. field surfaces were processed using a home-written MATLAB program that removes the background signal before the laser pulse (signal vs. magnetic field) and the intrinsic response of the cavity to the laser pulse (signal vs. time). The TR-EPR spectra shown in the main text were extracted from the surface at the maximum of the transient to avoid potential distortions arising from anisotropic relaxations: 1000 ns after the laser flash in all solvents, 1700 ns after the laser flash in WSCP.

### 2.3. Spectral Analysis

Simulations of triplet spectra have been performed using a home-written MATLAB program [54]. The program calculates the TR-EPR spectrum as superposition of spectra obtained for a uniform distribution of the orientation of the molecules. To account for MPS effects, the anisotropic orientational distribution of excited molecules is introduced. Fittings have been performed using the *esfit* routine from EasySpin combined with custom fitting functions [70]. Details of the calculation and of the theoretical basis for simulation are reported in [54,71] and in the Supplementary Materials.

### 2.4. Excitonic Coupling Calculation

The excitonic coupling between each pair of Chls *a* in the WSCP structure (PDB.ID 2DRE [31]) was calculated in the framework of the point dipole approximation. This approach, although simple when compared to more advanced methodologies, has been shown to be suitable for describing Chls Q_y_ − Q_y_ interactions in many systems [16,72,73]. The calculation was limited to the Q_y_ transitions of the four Chls *a*, varying the orientation of the transition dipole moments from the starting direction defined from the positions of the nitrogen atoms of the pyrrole rings A and C (axis *y* in Figure 1B), according to Madjet et al. [13]. A transition dipole moment of 4.67 D has been adopted for Chl *a* [16].

The point dipole coupling term of the *j*-th and *i*-th chromophores, $V_{ji}$ is calculated as:

$$V_{ij}=\frac{f_{1}}{4\pi \varepsilon_{0}}\left[\frac{\mu_{i}\cdot \mu_{j}}{R_{ij}{}^{3}}-3\frac{\left(\mu_{i}\cdot R_{ij}\right)\left(\mu_{j}\cdot R_{ij}\right)}{R_{ij}{}^{5}}\right]$$

where $\mu_{i,j}$ is the transition dipole moment vector, $R_{ij}$ the distance vector, $\varepsilon_{0}$ is the dielectric constant in vacuum, and $\varepsilon_{r}$ is the relative dielectric constant taken with a value of 2.40 (as derived from a refractive index value of 1.55 typical for protein environments [31]). The local field correction f_1_ has been treated with the sphere-cavity approximation ($f_{1}$ = $(\varepsilon_{r}$ + 2)/3).

The Hamiltonian describing the excitonic system is treated in terms of first-order perturbation theory:

$$H=\underset{i}{\sum }|i\rangle E^{0}\langle i|+\underset{i\neq j}{\sum }|j\rangle V_{ji}\langle i|$$

where the site energy ($E^{0}$) has been considered the same for the four pigments, adopting the previously determined value of 15,080 cm^−1^ [16].

Solving the Hamiltonian, by means of a home-written MATLAB program, it was possible to determine the eigenvalues and eigenvectors for the four excitonic states (named in the following M1, M2, M3, and M4 in the order of increasing energy). From the eigenvectors, directions and oscillator strengths (${\left|\mu_{M_{i}}\right|}^{2}$) of the four excitonic states were obtained [74]. All four Chls were considered a potential site of ^3^Chl *a* localization as the monomeric nature of ^3^Chl *a* in WSCP has been previously established spectroscopically [16,27] and the four binding sites are identical due to the D_2_ symmetry of the system (in Figure 2B, the example of the relative orientation between M3 TDM and the ZFS axes of ^3^Chl *a* localized on one of the four Chls is reported). For this reason, average values are reported in the following and in Tables S2 and S3.

## 3. Results and Discussion

In this work, we present the potential of the MPS methodology to gain quantitative information on the excitonic network among chromophores in the WSCP complex, employed as a paradigmatic chlorophyll-binding protein for the investigation of photosynthetic proteins, due to its defined symmetric structure. MPS provides a key link between the optical and magnetic resonance experiments. The spectral analysis allows quantitative determination of the relative orientation between the TDM, corresponding to the singlet state excited excitonic transition, and the ZFS principal axes of the triplet state, populated by inter-system crossing and localized on a single Chl *a* chromophore in WSCP.

**Figure 3:**
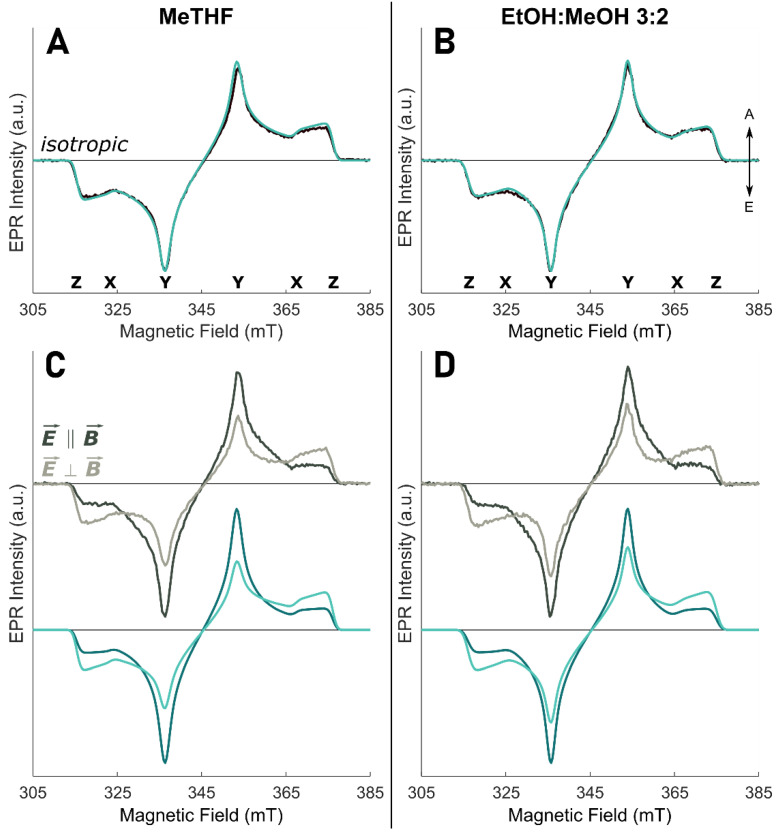
TR-EPR spectra (greyscale lines) and simulations (green lines) of Chl *a* in solvent. (**A**) MeTHF (λ = 661 nm), isotropic excitation; (**B**) EtOH:MeOH 3:2 (λ = 672 nm), isotropic excitation; (**C**) MeTHF (λ = 661 nm), MPS experiment; (**D**) EtOH:MeOH 3:2 (λ = 672 nm), MPS experiment. In MPS spectra: dark colours—laser polarized parallel to the magnetic field ($\vec{E}∥\vec{B}$); light colours—laser polarized perpendicular to the magnetic field ($\vec{E}⊥\vec{B}$ ). The arrows denote enhanced absorption (A) and emission (E); the positions of the principal ZFS components are indicated below the isotropic spectra. Spectra and simulations are normalized to the most intense spectral feature, keeping the intensity ratio between the two polarizations. All TR-EPR spectra are recorded at 80 K.

We initially performed TR-EPR, coupled to linearly polarized light excitation, on ^3^Chl *a* in two organic glasses in order to (i) compare the results of our MPS analysis to previously determined TDM orientation by different methods, (ii) check the sensitivity of the technique to the solvent dependence of the TDM, and (iii) directly determine the relative orientation between the TDM and ZFS axes for the monomeric Chl *a* components which form the tetra-chromophoric complex. The two solvents have been selected for their propensity to form transparent glasses. MeTHF is characterized by a low dielectric constant, similar in value to those of protein matrixes, whereas the EtOH:MeOH mixture has a much higher one. The results are reported in Figure 3.

The excitation around the maximum of the Q_y_ (0-0) absorption band, at 661 nm for MeTHF and 672 nm for EtOH:MeOH, provides selective Q_y_ excitation since the Q_x_ transition has negligible absorption at these wavelengths. Figure 3A,B displays the isotropically excited TR-EPR spectrum in the two different glassy matrices at 80 K and the corresponding simulations. In order to fulfill isotropic excitation conditions, the TR-EPR spectrum is obtained as the sum of the experimental spectrum recorded after excitation with polarization of the light parallel to the external magnetic field and twice the spectrum obtained with perpendicular polarization, in analogy to optical polarization measurements. The simulation parameters, reported in Table 1, are in good agreement with previous work performed without linearly polarized light for excitation [27]. They have been used as fixed parameters in the simulations of the MPS experiments in the following.

The TR-EPR spectra, obtained exciting with light polarized either parallel or perpendicular to the external magnetic field, are reported in Figure 3C,D. Since the experimental set-up assured the same excitation conditions, the spectra are displayed with their relative intensities and show an evident MPS effect, with enhanced Y canonical transitions in the parallel spectrum and enhanced X and Z canonical transitions in the perpendicular spectrum, in both glass matrices. The simulations, based on common parameters for the two excitation modes, exhibit the correct orientational effects and also reproduce the relative intensities of the parallel and perpendicular configuration. The presence of E-strain, due to an inhomogeneous coordination environment or molecular distortions, affects the lineshape in correspondence of the X and Y transitions, precluding an accurate simulation of the lineshape between the two turning points.

The key parameters of the spectral simulations are the angles ω_m_ and φ_m_ as defined in Figure 2A, while the ZFS parameters (D and E) and the relative triplet population rates are derived from the isotropically-excited spectrum (see Table 1). The angle ω_m_ is kept fixed at 90° to fulfil the condition that Q_y_ and the X, Y ZFS axes lay in the macrocycle plane. The value obtained for φ_m_ is in excellent agreement with those obtained by Vrieze et al. in the LD-ODMR experiments [65] and confirm a solvent-dependent configuration, where the Q_y_ TDM and the in-plane triplet axes are not collinear, in contrast to other porphyrin derivatives [54,75]. Therefore, we can safely state that MPS is a sensitive method for the determination of the orientation of the TDM in the ZFS frame. In particular, based on the selection of specific excitation wavelengths, for ^3^Chl *a* in vitro the TDM orientation of the Q_y_ transition is derived, while for the ^3^Chl *a* in WSCP the TDM corresponding to a Mi (i = 3 and 2) excitonic transition is obtained. In both cases, the TDM orientation is given with respect to the ZFS principal axes (X, Y, and Z), as experimentally derived.

**Figure 4:**
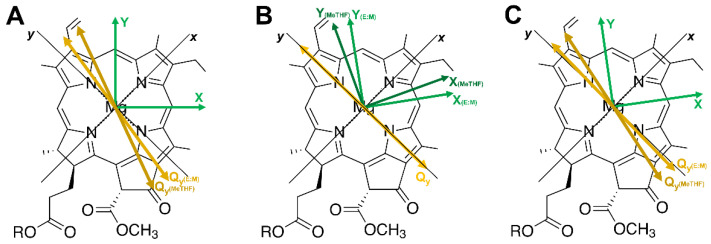
Chl *a* structure is shown with the transition dipole moment of the Q_y_ transition (yellow), the molecular axes (black), and the X and Y ZFS axes. (**A**) Q_y_ TDM (dark yellow) has been rotated accordingly to the φ_m_ determined for Chl *a* in MeTHF and EtOH:MeOH 3:2, while keeping the ZFS frame (green) as in Figure 2. (**B**) X and Y ZFS axes (dark green) have been rotated accordingly to the φ_m_ determined for Chl *a* in MeTHF and EtOH:MeOH 3:2, while keeping the Q_y_ TDM along the *y* axis. (**C**) X and Y ZFS axes (green) have been rotated by 8° and the Q_y_ TDM (dark yellow) has been rotated accordingly to the φ_m_ determined for Chl *a* in MeTHF and EtOH:MeOH 3:2.

The MPS experiment allows the determination of the relative orientation of the TDM and ZFS axes, but not their localization in the molecular frame (*x*, *y* and *z*). Thus, our results do not distinguish between the two different configurations, as illustrated in Figure 4A,B. One possibility is obtaining the Q_y_ orientation based on the assignment of ZFS axes directions, as derived from triplet-state ENDOR experiments [66,76] (see Figure 4A). Alternatively, the Q_y_ TDMs can be fixed in the molecular frame as parallel to the molecular *y* axis, consequently assigning the triplet ZFS directions (see Figure 4B). Although the two possible configurations cannot be discerned, MPS experiments introduce angular constraints and clearly demonstrate that the assignment of the Q_y_ TDM along the molecular *y* axes as well as that of the ^3^Chl *a* ZFS tensor axes of ^3^Chl *a* along the methine protons, based on spectroscopic or in silico investigation, should be considered with caution.

The MPS results obtained for the Chl *a* pigment in glassy matrix were used to guide simulations of the corresponding experiments performed on the ^3^Chl *a* in the tetrameric complex and gain information on the relative orientation between the excitonic TDMs and the ZFS axes of the Chl carrying the triplet state in WSCP (the corresponding angles ω_ex_ and φ_ex_ are reported in Figure 2B).

**Figure 5:**
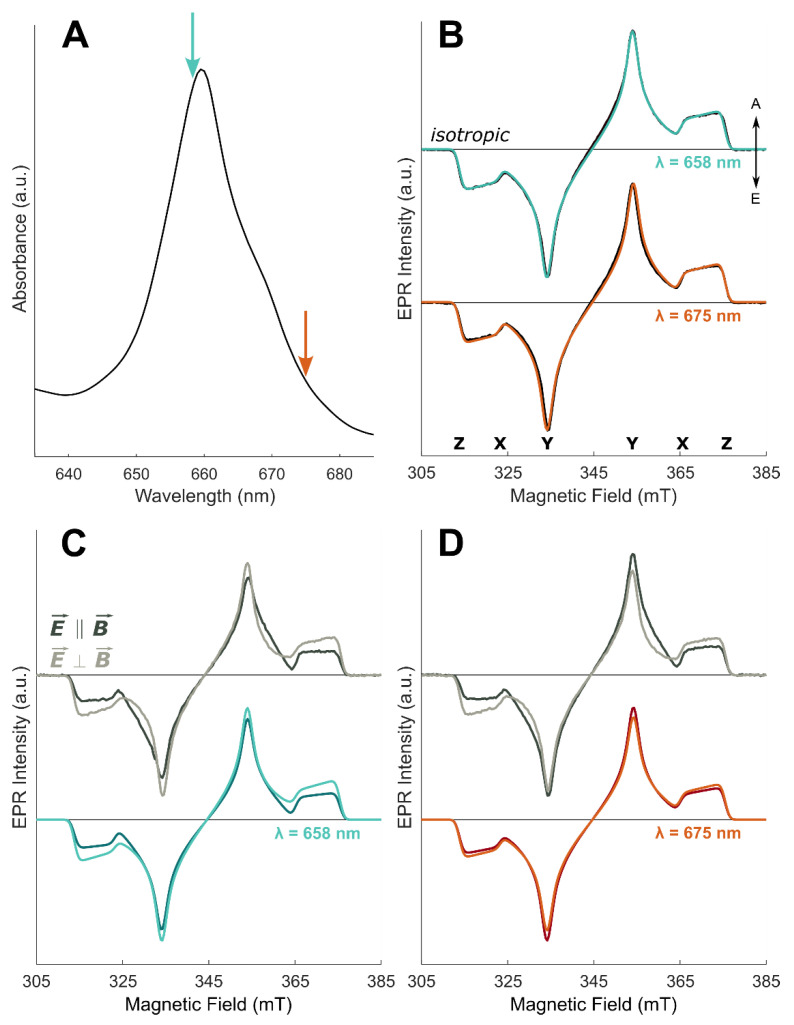
Spectra (greyscale lines) and simulations (coloured lines) of Chl *a* in WSCP. (**A**) Low-temperature UV-Vis absorption spectrum of WSCP in the Q-bands (adapted from [16]), temperature 1.8 K; the coloured arrows point to the wavelength used to obtain the TR-EPR spectra: green λ = 658 nm; red/orange λ = 675 nm. (**B**) TR-EPR spectra with isotropic excitation (greyscale lines) and simulations (coloured lines). (**C**,**D**) MPS TR-EPR spectra (greyscale lines) and simulations (coloured lines): dark colours—laser polarized parallel to the magnetic field ($\vec{E}∥\vec{B}$); light colours—laser polarized perpendicular to the magnetic field ($\vec{E}⊥\vec{B}$ ). The arrows denote enhanced absorption (A) and emission (E); the positions of the principal ZFS components are indicated below the isotropic spectra. Spectra and simulations are normalized to the most intense spectral feature keeping the intensity ratio between the two polarizations. All TR-EPR spectra are recorded at 80 K.

The isotropically-excited TR-EPR spectra of ^3^Chl *a* in WSCP at 80 K, with excitation at two different wavelengths, are shown in Figure 5B. Moving along the Q_y_ absorption region allows the selection of different excitonic contributions. Based on calculations, shown in Figure 1C, M2 is expected to be excited on the red-shifted shoulder, observed in Figure 5A. The ZFS parameters and the relative population rates obtained from the simulation of the spectra are, within error, identical to those obtained previously by pulse and time-resolved EPR at X-band [27] and do not vary significantly with the excitation wavelength (see Table 2). The magnetic parameters are also very similar to those of ^3^Chl *a* in the solvent glass, confirming localization of the triplet exciton on a monomeric Chl *a* in WSCP, as demonstrated previously by ENDOR spectroscopy [27,46].

The MPS spectra at the two different excitation wavelengths are reported in Figure 5C,D. The spectra show MPS effects that differ for the two selected wavelengths and also from those found for the ^3^Chl *a* in glassy matrix. Qualitatively, the differences with the excitation wavelength point out a different orientation of the excitonic TDM with respect to the ZFS frame or contributions of different excitonic transitions, as expected from calculations.

The simulations of the MPS experiments in WSCP are based on angular parameters obtained from the crystallographic structure [31] in the framework of a strong excitonic interaction in the excited singlet state, which is lost in the corresponding triplet state. Due to the symmetry of the WSCP tetrameric complex, the four Chl binding sites are equivalent and the triplet state is localized on any of the Chl *a* monomers in the protein with the same probability. As pointed out in the previous sections, MPS experiments on ^3^Chl *a* in a glassy matrix demonstrate that the orientation of the Q_y_ TDM could deviate from the molecular *y* axis. Moreover, the influence of the environment on the Q_y_ TDM location in the molecular plane, which can be attributed to solvent polarity, hydrogen bonding, and/or molecular distortion effects, has also been highlighted. For this reason, calculation of the excitonic TDMs required an angular scanning of the orientation of the Q_y_ TDM and the ZFS axes in the molecular frame. Initially, calculations were performed assuming that the Q_y_ TDM is collinear to the molecular *y* axis and the in-plane ZFS axes are along the methine groups (Figure 1B), as defined by Lendzian et al. [66], but with the uncertainty deriving from the orientational selection of ENDOR spectroscopy (i.e., ±10° from the molecular *y* axis). From this starting point, calculations were repeated for different orientations of the Q_y_ TDM and for gradual rotation of the ZFS frame (see Figure S5), in a range that comprises the angular parameters found for ^3^Chl *a* in glassy matrix and those reported in the literature [8,10,11,12,13,14,15]. Details are described in the Materials and Methods and the full set of data is summarized in the SI.

We first analysed the EPR dataset obtained at 658 nm, near the maximum of the excitonic absorption band. At this wavelength, we expect only a contribution from the M3 transition, since its oscillator strength is much higher than the oscillator strength corresponding to M4 (within all the selected angle for the Q_y_ TDM based on magnetophotoselection in vitro), the only other transition close in frequency [16] (see Table S1). We verified that, due to symmetry reasons, displacements of the Q_y_ TDM of the Chl *a* monomers with respect to the principal triplet axes, in a range of ±10°, does not significantly change the orientation of the M3 excitonic TDM in terms of the angles ω_ex_ and φ_ex_ (defined in Figure 2B). On the other hand, rotations of the ZFS axes do affect the value of φ_ex_. The simultaneous fitting of the TR-EPR spectra (Figure 3C), for the parallel and perpendicular configurations, was performed fixing the value of ω_ex_ to 76°, as calculated in the frame of the excitonically coupled Chl *a* tetramer. An optimal φ_ex_ angle of 30° was obtained as indicated in Table 2, where all the simulation parameters are also reported. These angular parameters allow us to precisely determine the orientation of ZFS axes in the molecular frame: a rotation of 8° with respect to the axis passing through the methine groups has been found(see Figure 4C). Assignment of the ZFS tensor directions in the molecular frame is an important task to be fulfilled for exploiting the full potential of orientation-selective triplet-state EPR techniques [77,78]. As a further result, fixing the ZFS directions, this leads to a final orientation of the Q_y_ TDM axes in the Chl *a* monomers, which translated to MeTHF and EtOH:MeOH mixture gives a deviation of 12° and 3° from the molecular *y* axis, respectively. The assumption that the ZFS tensor directions are the same in the solvents and in WSCP is based on the fact that the ZFS axes orientations are not particularly affected by the environment, as demonstrated by the comparative ENDOR investigations on ^3^Chl *a* in glassy matrix, in WSCP, as well as in photosynthetic reaction centres [27,46,66,78]. Thus, exploiting the excitonic interaction, we were also able to give the orientation of the Chl *a* Q_y_ TDM, resulting in it being slightly rotated with respect to the molecular *y* axis.

The TR-EPR spectra obtained by photoexcitation at 675 nm can be considered the sum of two contributions, one still deriving from the excitation of the M3 transition and a second one due to the excitation of either the M1 or M2 transitions. In general, considering all the four excitonic transitions, the rotation of the Q_y_ TDM of the monomer in the molecular plane does not alter the orientation of the resulting excitonic TDMs (less than 1° in the investigated range of ±10°), but does affect the oscillator strengths. In particular, a rotation of Q_y_ can reverse the order of the oscillator strengths of M1 and M2 (see Table S1). For this reason, as a second contribution to the experimental MPS effect, the relative orientations of the M1 and the M2 TDMs with respect to the triplet frame were alternatively considered as angular parameters to simulate the TR-EPR spectra obtained at 675 nm. The polar angles were calculated, for both transitions, fixing the monomer Q_y_ TDM along the molecular *y* axis and rotating the in-plane ZFS axes of 8°, as obtained for the simulation of the TR-EPR spectra at 658 nm. The complete angular scanning is reported in the SI, together with frequencies and oscillator strengths of the excitonic transitions. Simulations of the corresponding triplet TR-EPR spectra, in the parallel and perpendicular excitation mode, demonstrates that the orientation of the M1 TDM (ω_ex_ = 42°, φ_ex_ = 44°) is incompatible with the MPS effects recorded in WSCP (see Figure S6B,C), while for M2 (ω_ex_ = 52°, φ_ex_ = 72°) a satisfactory agreement can be obtained. A fitting of M2 and M3 relative contributions, keeping their orientation fixed at the values obtained for the first data set, was successively performed, as shown in Figure 5D.

We have therefore demonstrated that the two independently measured triplet-state spectra, excited with light polarized parallel and perpendicular to the EPR magnetic field, can be satisfactorily simulated with the same set of angular parameters, and that an excellent agreement is found with the angular parameters calculated in the point-dipole approximation for the four Chls excitonically coupled and arranged in two open-sandwich dimers, based on the X-ray structure of the pigment cluster. The localization of the triplet state on a single chromophore is an important prerequisite for a correct and effective characterization of the excitonic system, as demonstrated here in the case of WSCP. This is a common case since excitonic interactions in the triplet state are in most cases negligible as they depend exclusively on exchange integrals and are therefore of reduced size compared to the corresponding interaction involving singlet states, whose dependence on additional long-range Coulomb integrals ensures a significant coupling in a wide distance range exceeding 1 nm [79].

Since a simplified excitonic model, which considers the interactions between the two Chl *a* dimers negligible (see Figure 1A), was used to satisfactorily reproduce the absorption and circular dichroism spectra of WSCP [14,80], we have verified its appropriateness to describe the MPS experiments. The two excitonic states of each of the two identical dimers are named MH and ML. The higher energy MH transition is characterized by a TDM with the same orientation to the one corresponding to M3 transition, whereas in the case of the lower energy ML transition the TDM orientation differs from those corresponding to both M1 and M2, resulting in a specific set of polar angles with respect to the Chls *a* ZFS frames (ω_ex_ = 30°, φ_ex_ = 72°). As described in detail in the SI, while the MH transition is compatible with the triplet TR-EPR spectra at 658 nm, the polar angles corresponding to the ML transition preclude the simulation of the TR-EPR spectra at 675 nm (see Figures S6C and S7C). This result confirms that in the description of the excitonic states of WSCP a tetrameric model is required, as previously suggested on the basis of ODMR results [16]. Moreover, this outcome highlights the sensitivity of MPS to variations of the geometry of the multi-chromophoric system and the excitonic network, making it an experimental technique very well suited to investigate these systems.

## 4. Conclusions

MPS is an important methodological tool for deriving orientation parameters as demonstrated in the comparative investigation on ^3^Chl *a* monomer in glassy matrix and embedded in the WSCP complex. From the simulation of the TR-EPR spectra, excited with light polarized parallel and perpendicular to the magnetic field, the direction of the TDM in the frame of the principal triplet axes were determined with high accuracy. The information on the angular parameters is crucial to gain insight into the excitonic structure of the protein complex, going beyond the chromophore organization provided by the crystallographic structure. This renders MPS complementary to other spectroscopic techniques to contribute to a complete picture of structure-function relationships in electronic states other than the ground state. In the present work, this approach has confirmed that a tetrameric model is required to properly describe the interactions among Chls and has provided important details on the excitonic structure of WSCP. Furthermore, for the first time, precise information on the orientation of the ZFS axes of ^3^Chl *a* in the molecular frame has been accessible due to the symmetry of the cluster and to the localization of the triplet state.

The application of this high-resolution methodology to antenna complexes would provide key information for structure-based quantitative calculation of excitonic couplings between photosynthetic pigments. As a design strategy of nature, excitonic interactions in photosynthetic light-harvesting complexes are used to expand the spectral cross-section for light absorption and ensure a considerably faster and directional excitation energy transfer. Although WSCP is not involved in the photosynthetic process, it is considered an ideal model system to investigate the detailed nature of the pigment-protein and pigment-pigment interactions at the heart of this process, due to its relatively simple structure and to the possibility to modify the number and molecular structure of the bound chromophores [27,81,82,83,84]. Unravelling the details of excitonic delocalization is a fundamental requisite to improve artificial photosynthetic systems based on multi-chromophore assemblies [85,86], and high-resolution MPS represents an effective spectroscopic tool giving important constraints to verify the applicability of theoretical models to describe these complex systems.

## Figures and Tables

**Table 1:** Simulation parameters used in the fitting of Chl *a* MPS spectra in glassy matrix. ZFS parameters (D, E), ±0.1 mT; triplet sublevel populations (p_x_, p_y_, p_z_), ±0.01; angles defining the orientation of the transition dipole moment relative to the ZFS axes system (ω_m_ and φ_m_, defined in Figure 2), ±2°; percentage of the polarized contribution (c_P_), ±1%. Details of the parameters are reported in the Supporting Information.

Solvent	|D| (mT)	|E| (mT)	p_x_	p_y_	p_z_	ω_m_ (°)	φ_m_ (°)	c_P_ (%)
**MeTHF**	30.4	4.3	0.31	0.56	0.13	90	65	63
**EtOH:MeOH 3:2**	29.5	3.8	0.31	0.57	0.12	90	54	64

**Table 2:** Simulation parameters used in the fitting of WSCP MPS spectra. Excitation wavelength (λ); ZFS parameters (D, E), ±0.1 mT; triplet sublevel populations (p_x_, p_y_, p_z_), ±0.01; angles defining the orientation of the ZFS axis system relative to the transition dipole moment (ω_ex_ and φ_ex_, defined in Figure 2); percentage of the polarized contribution (c_P_), ±1%; percentage of each spectral component (W), ±1%. Details of the parameters are reported in the Supporting Information.

λ (nm)	|D| (mT)	|E| (mT)	p_x_	p_y_	p_z_	ω_ex_ (°)	φ_ex_ (°)	c_P_ (%)	W (%)
**658**	30.9	3.65	0.29	0.59	0.12	76	30	57	100
**675**	30.9	3.65	0.29	0.59	0.12	52	72	57	64
	30.9	3.65	0.29	0.59	0.12	76	30	57	36

## Data Availability

Data are available from the corresponding author upon request.

## Supplementary Materials

The following supporting information can be downloaded at: https://www.mdpi.com/article/10.3390/molecules27123654/s1, Description of the technique; Figures S1–S7; Tables S1–S3. References [87,88] are cited in the supplementary materials.

## Sample Availability

Samples are available from the corresponding author upon request.
