# Supplementary material for: Magnetophotoselection in the Investigation of Excitonically Coupled Chromophores: The Case of the Water-Soluble Chlorophyll Protein
Source: Molecules. 2022 Jun 7;27(12):3654. doi: 10.3390/molecules27123654 (PMC9227413; doi:10.3390/molecules27123654)
Supplement: Supplementary file 1 [file molecules-27-03654-s001.zip › molecules-1735860-supplementary.pdf]

molecules

MDPI

Supplementary Materials

Magnetophotoselection in the Investigation of Excitonically Coupled Chromophores: The Case of the Water-Soluble Chlorophyll Protein

Susanna Ciuti [1,†], Alessandro Agostini [1,2,†], Antonio Barbon [1], Marco Bortolus [1], Harald Paulsen [3], Marilena Di Valentin [1,*] and Donatella Carbonera [1,*]

[1] Department of Chemical Sciences, University of Padova, via Marzolo 1, 35131 Padova, Italy; susanna.ciuti@phd.unipd.it (S.C.); alessandro.agostini@umbr.cas.cz (A.A.); antonio.barbon@unipd.it (A.B.); marco.bortolus@unipd.it (M.B.)

[2] Biology Centre, Czech Academy of Sciences, Institute of Plant Molecular Biology, Branišovská 1160/31, 370 05 České Budějovice, Czech Republic

[3] Institute of Molecular Physiology, Johannes Gutenberg-University of Mainz, Johann-Joachim Becher-Weg 7, 55128 Mainz, Germany; paulsen@uni-mainz.de

* Correspondence: marilena.divalentin@unipd.it (M.D.V.); donatella.carbonera@unipd.it (D.C.); Tel.: +39-0498275139 (M.D.V.); +39-0498275144 (D.C.)

† These authors contributed equally to this work.

Index:

	Page
Time Resolved EPR (TR-EPR) and Magnetophotoselection (MPS) of Triplet States *including* Figures S1-S4	2–6
Tables S1-S2	7
Figure S5	8
Nature of the second excitonic transition at 675 nm *including* Table S3, Figures S6-S7	9–11
References	12

Time Resolved EPR (TR-EPR) and Magnetophotoselection (MPS) of Triplet States

We start this section by warning the reader that we are going to use similar terminology with rather different physical meaning; in particular, we are going to use orientational polarization, polarization (of population) of the triplet sublevel or spin polarization, and laser polarization. The concepts will be gradually introduced and explained.

We briefly introduce basic concepts of TR-EPR of triplet states of organic molecules [87,88].

Photoexcited states of organic dyes are generally characterized by the presence of two unpaired electrons. The triplet states of these systems are defined within the coupled representation, and they are characterized by the spin quantum number S=1. In the hypothetical case of absence of electron-electron interactions, the three degenerate sublevels (distinguished by the values of m_s=+1, 0, −1) are degenerate, but the degeneracy can be removed by application of an external magnetic field because of the Zeeman interaction $H_{zee} = \mu_B \boldsymbol{S} \cdot \underline{\boldsymbol{g}} \cdot \boldsymbol{B}$, where μ_B is the Bohr magneton, $\boldsymbol{S}$ the spin operator, $\underline{\boldsymbol{g}}$ the g-tensor and $\boldsymbol{B}$ the magnetic field. In this system, only two transitions can be stimulated (allowed transitions) by microwave irradiation: $m_s = -1 \leftrightarrow m_s = 0$ and $m_s = 0 \leftrightarrow m_s = +1$ at resonance conditions (see fig. S1, left). Resonance conditions is the combination of microwave frequencies (for X-band instruments typical values are near 9.5 GHz) and magnetic fields for which $\Delta E = h\nu$, where ΔE is the energy difference between consecutive states (depending on the magnetic field), and ν is the microwave frequency. These conditions can be searched for by sweeping the magnetic field while measuring the absorption of microwaves, kept at a fixed frequency. Because the energy difference between two consecutive levels is the same ($\Delta E = \mu_B g B$, g is the effective g-value), for this simple system, we can observe only a single line.

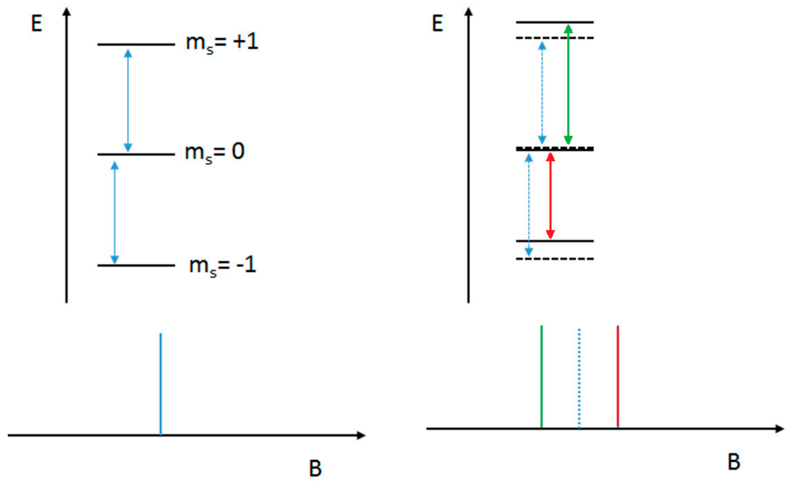

Figure S1. effect of the magnetic field (B) on the triplet sublevels in the case of no ZFS interaction (left) and with ZFS interaction (right). Resonance conditions lead to two degenerate (left) or to non-degenerate transitions (right).

In regular triplet states of organic molecules, rarely the energy difference between the levels is the same, as the two electrons interact through the so-called *zero-field splitting* (ZFS) magnetic interaction. The consequence is that the zero-field degeneracy of the triplet sublevels is removed, and in the magnetic field the energy difference between consecutive levels is not the same. Therefore, the two allowed transitions occur at different magnetic fields (see fig. S1, right) and two bands result separated in the EPR spectrum; the line separation depends on the strength of the interaction.

In general, the spin Hamiltonian for ZFS interaction is $H_{ZFS} = \boldsymbol{S} \cdot \underline{\boldsymbol{D}} \cdot \boldsymbol{S}$; $\underline{\boldsymbol{D}}$ is the dipolar tensor, whose principal vales, D_x, D_y and D_z, are the opposite of the energies of the three sublevels at zero field (X, Y and Z, eigenvalues of H_{ZFS}). Because $\underline{\boldsymbol{D}}$ is a traceless

tensor, only two parameters are needed to describe the three ZFS eigenvalues: D, related to the (absolute) larger energy value ($D = -3Z/2$), and E that is related to the rhombicity of the tensor ($E = (Y - X)/2$).

Let's now consider the spectrum obtained for powder-like samples.

The ZFS Hamiltonian accounts for a dipolar interaction that varies with the orientation of the system with respect to an observation orientation (in EPR it is the orientation of the magnetic field). For a uniform distribution of molecules, like in a glassy frozen diluted solution, we expect to observe pairs of lines for the different orientation of the molecules. The maximum separation between the lines is attributed to molecules oriented with the Z-principal direction of the dipolar tensor along the magnetic field.

Because triplet states are transient species, it is necessary to monitor the EPR signal of the system as a function of time following a laser pulse, for different values of the magnetic field. In the Time Resolved EPR (TR-EPR) spectra shown in this work, the spectra report the EPR intensity at a fixed minimum delay (determined by the instrument time response) after the laser excitation.

A typical TR-EPR spectrum of Chl *a* is reported in Fig. S2.

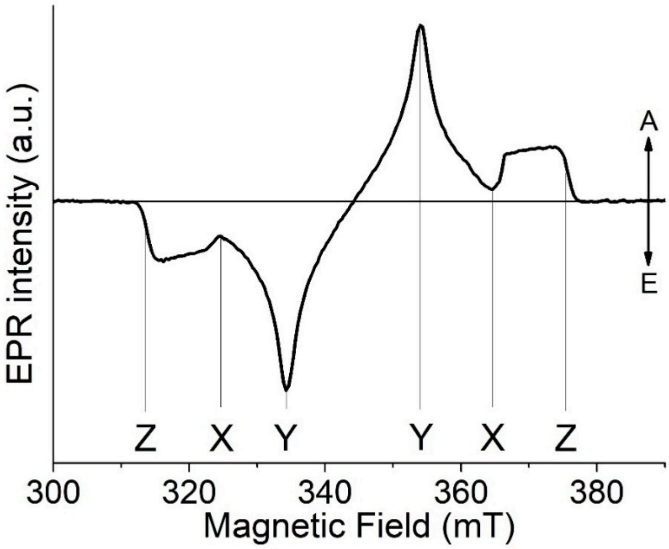

Figure S2. TR-EPR spectrum of ^{3}Chl *a* in WSCP dissolved in a glassy frozen solution at low temperature and isotropic excitation. The arrows denote enhanced absorption (A) and emission (E); the positions of the principal ZFS components are indicated below the spectrum.

There are three pairs of features that are observed in the spectrum, marked with lines. They refer to the pair of transitions attributed to molecules oriented with the principal directions (indicated in the figure) of the dipolar tensor along the magnetic field.

It is possible to note that the spectrum is spin polarized: intensities of the transitions are in enhanced absorption or in emission, as indicated by the arrows in figure S2. Spin polarization is an indication that the population of the triplet sublevels occurred by a spin selective mechanism, in this case a regular intersystem crossing (ISC), leading to a non-Boltzmann population of the states. In the pair of transitions, the two lines are with opposite polarization (A/E or E/A)

The simulation of these powder spectra, as described in ref. [54,71], can be obtained as the superposition of the spectra obtained from all possible relative orientations of the magnetic field and the molecules. Two polar angles α, β are sufficient to express the orientation of the magnetic field in the ZFS frame, taken as reference. As previously described, the spectrum obtained from molecules with a particular orientation is composed of two bands located at two $B_{\text{res}}(\alpha, \beta)$ values (relative to the two triplet allowed transitions, +/-); resonance fields can be obtained after diagonalization of the spin Hamiltonian

of the system, from the energy difference between consecutive levels. In real systems, each transition possesses a proper Gaussian/Lorentzian lineshape $G(B_{\text{res}}(\alpha,\beta) - B)$ and a finite linewidth. The intensity of the two bands is proportional to the population difference between two consecutive states $P_{\pm}(\alpha,\beta,B)$; the populations are determined by ISC.

Therefore, for an isotropically excited sample, the calculation runs as:

$$I(B) \quad = \sum_{+/-} \int_0^{2\pi} \int_0^{\pi} G(B_{\text{res}}(\alpha,\beta) - B) \cdot P_{\pm}(\alpha,\beta,B_0) \cdot \sin\beta \, d\alpha \, d\beta \qquad \text{(eq. S1)}$$

A different result is obtained when optical polarized light is used, because the orientation of the electric component of the electromagnetic electric field operates a selection of the excited molecules based on their orientation. We call the direction of the electric field of the laser beam the laser polarization. The consequence in the TR-EPR spectrum is that only molecules oriented in a particular way may contribute to the spectrum. This is the so-called a magnetophotoselection effect.

Magnetophotoselection of triplet states potentially can be observed in most solid-states systems, having diluted dye molecules or cluster of dyes, provided that a) the molecules/clusters have a well-defined transition dipole moment, TDM b) polarized light is used for the excitation c) molecules/clusters form triplet states.

Let's then considered a typical system composed of a diluted solution of a dye molecule, cooled down to form a transparent glass. Let's also use a polarized laser beam for excitation at a wavelength within the absorption spectrum the molecule.

If conditions a)-c) are all fulfilled, excitation of the solution leads to a transient population of a selection of excited molecules; indeed, the probability for the molecules to be excited depends on the square of the angle between the TDM (see figure S3). The maximum probability of absorption is for molecules having $\vartheta = 0^0$, no absorption is possible for molecules with $\vartheta = 90^0$. In absence of scattering events from the solution that can reduce or quench the laser polarization, they represent a small fraction of molecules and are characterized by a rough collinearity of the direction of their TDMs (an orientational polarization), which is defined by the laser polarization.

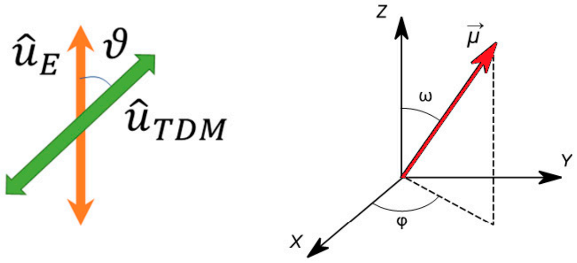

Figure S3. (left) relative orientation of the transition dipole moment and the direction of optical polarization of the excitation light, determined by the direction of the electric field. (right) orientation of the Transition Dipole Moment (TDM) in the ZFS frame.

We can then introduce an orientational distribution function, $p(\vartheta)$, which describes to the probability to find an excited molecule with a TDM oriented for a particular value ϑ. This will be $p(\vartheta) \propto \left(\hat{u}_{\mu} \cdot \hat{u}_{E,z}\right)^2 = cos^2\,\vartheta$

This orientational distribution is preserved when the molecules undergo to the singlet-to -triplet interconversion in the absence of specific relaxation processes. This distribution function needs to be included in the spectrum calculation as a factor weighting the contribution to the spectrum of a particular direction.

Magnetophotoselection experiments are normally used to determine the orientation of the TDM within the ZFS frame; the orientation of the TDM is expressed by the polar angles ω, φ (see figure S3, right). For simplicity, let' assume that the TDM is oriented along the Y-principal direction ($\omega = 90°, \varphi = 90°$). If the laser polarization is parallel to the external magnetic field, excitation will produce an orientational distribution with

excited molecules oriented with their TDM along the field. After ISC, then, there will be a selection of molecules in the triplet state having all Y-principal direction oriented along the magnetic field, and in the TR-EPR spectrum we'll obtain a dominance of the intensity of the Y-components (see Figure S4). On the contrary, if laser polarization is perpendicular to the magnetic field, the X-components will be depleted, and the Y- and Z-components will be enhanced.

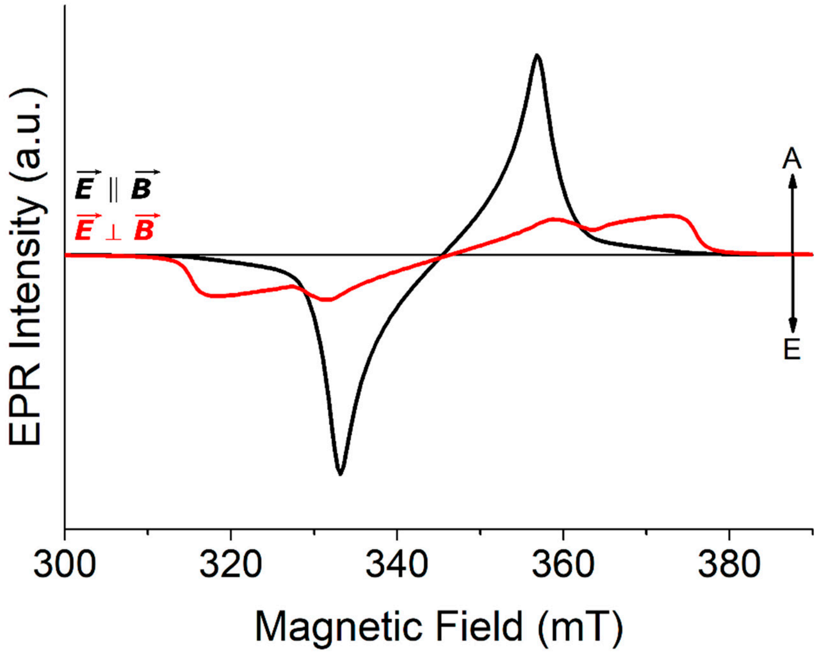

Figure S4. Simulation of a TR-EPR spectrum in the presence of magnetophotoselection effects. The parameters for the ZFS values and the populations are taken from Table 1 in the main text. Spectra with *par* and *perp* polarization of the laser polarization with respect to the magnetic field are displayed. It is assumed that the TDM is along the Y component ($\omega = 90°, \varphi = 90°$).

A careful and simultaneous simulation of the spectra obtained with parallel (*par*) or perpendicular (*perp*) polarization with respect to the magnetic field (and ω, φ as free parameters) allows obtaining the orientation of the TDM in the molecule.

The calculation of the spectrum requires the introduction in eq. S1 of the orientational distribution function

$p_{\omega,\varphi}^{\text{par/perp}}(\alpha,\beta,\gamma) = \left[\hat{u}_\mu(\omega,\varphi) \cdot \hat{u}_E(\alpha,\beta,\gamma)\right]^2/N$ as produced by the *par* or *perp* excitation (three Euler angles are now required to express the orientations of both the laser polarization and of the magnetic field).

The calculation of the spectra with magnetophotoselection effects are then

$$I(B)^{\text{par/perp}} = \sum_{+/-} \int_0^{2\pi} \int_0^{\pi} G(B_{\text{res}}(\alpha,\beta) - B) \cdot P_\pm(\alpha,\beta,B) \cdot \left(\int_0^{2\pi} p_{\omega,\varphi}^{\text{par/perp}}(\alpha,\beta,\gamma)\, d\gamma\right) \sin\beta \, d\alpha \, d\beta \quad \text{(eq. S2)}$$

Because experimentally it was impossible to avoid a small depolarization of the excitation light (for example because of the presence of small cracks in the glass, or because of reflections inside the EPR cavity), in the calculation we allowed a contribution to the spectrum as due to unpolarized component, therefore the lineshape of the experimental profile was calculated as

$$I(B)_{TOT} = C_P \cdot I(B)^{\text{par/perp}} + (100 - C_P) \cdot I(B) \quad \text{(eq. S3)}$$

where C_P is the % of polarized component.

Table S1. Calculated energies (E_{Mi}) and transition dipole moment square moduli ($|\mu_{Mi}|^2$) of the four excitonic transitions for WSCP (see figure 2A). Input parameters: transition dipole moment (μ_0) 4.6 Debye; site energy (E^0) 15080 cm^{-1}; relative dielectric constant (ε_r) 2.40.

	M1		M2		M3		M4	
Rot Qy	E_{M1} [cm^{-1}]	$\lvert\mu_{M1}\rvert^2$ [D^2]	E_{M2} [cm^{-1}]	$\lvert\mu_{M2}\rvert^2$ [D^2]	E_{M3} [cm^{-1}]	$\lvert\mu_{M3}\rvert^2$ [D^2]	E_{M4} [cm^{-1}]	$\lvert\mu_{M4}\rvert^2$ [D^2]
0°	14959	0.88	14989	4.83	15155	81.53	15218	0.01
+5°	14955	0.16	14991	7.80	15157	79.27	15217	0.00
+10°	14953	0.02	14994	11.35	15158	75.86	15215	0.00
−5°	14965	2.14	14989	2.51	15150	82.56	15215	0.02
−10°	14974	3.89	14991	0.96	15145	82.34	15210	0.04

Table S2. Dependence of the ω_{ex} and φ_{ex} angles for the four WSCP excitonic transitions from rotations of the monomeric TDM axis (Q_y transition, β) and of the X and Y ZFS axes around the molecular *z* axis (α). For a scheme of the axes and the angles see Figure S5, below.

		M1		M2		M3		M4	
β	α	ω_{ex}	φ_{ex}	ω_{ex}	φ_{ex}	ω_{ex}	φ_{ex}	ω_{ex}	φ_{ex}
0°	0°	42 ± 3	36 ± 1	52 ± 4	64 ± 2	76 ± 4	38 ± 1	72 ± 16	39 ± 19
	+1°	42 ± 3	37 ± 1	52 ± 4	65 ± 2	76 ± 4	37 ± 1	72 ± 16	38 ± 19
	+2°	42 ± 3	38 ± 1	52 ± 4	66 ± 2	76 ± 4	36 ± 1	72 ± 16	37 ± 19
	+3°	42 ± 3	39 ± 1	52 ± 4	67 ± 2	76 ± 4	35 ± 1	72 ± 16	36 ± 19
	+4°	42 ± 3	40 ± 1	52 ± 4	68 ± 2	76 ± 4	34 ± 1	72 ± 16	35 ± 19
	+5°	42 ± 3	41 ± 1	52 ± 4	69 ± 2	76 ± 4	33 ± 1	72 ± 16	34 ± 19
	+6°	42 ± 3	42 ± 1	52 ± 4	70 ± 2	76 ± 4	32 ± 1	72 ± 16	33 ± 19
	+7°	42 ± 3	43 ± 1	52 ± 4	71 ± 2	76 ± 4	31 ± 1	72 ± 16	32 ± 19
	+8°	42 ± 3	44 ± 1	52 ± 4	72 ± 2	76 ± 4	30 ± 1	72 ± 16	31 ± 19
	+9°	42 ± 3	45 ± 1	52 ± 4	73 ± 2	76 ± 4	29 ± 1	72 ± 16	30 ± 19
	+10°	42 ± 3	46 ± 1	52 ± 4	74 ± 2	76 ± 4	28 ± 1	72 ± 16	29 ± 19
	−1°	42 ± 3	35 ± 1	52 ± 4	63 ± 2	76 ± 4	39 ± 1	72 ± 16	40 ± 19
	−2°	42 ± 3	34 ± 1	52 ± 4	62 ± 2	76 ± 4	40 ± 1	72 ± 16	41 ± 19
	−3°	42 ± 3	33 ± 1	52 ± 4	61 ± 2	76 ± 4	41 ± 1	72 ± 16	42 ± 19
	−4°	42 ± 3	32 ± 1	52 ± 4	60 ± 2	76 ± 4	42 ± 1	72 ± 16	43 ± 19
	−5°	42 ± 3	31 ± 1	52 ± 4	59 ± 2	76 ± 4	43 ± 1	72 ± 16	44 ± 19
	−6°	42 ± 3	30 ± 1	52 ± 4	58 ± 2	76 ± 4	44 ± 1	72 ± 16	45 ± 19
	−7°	42 ± 3	29 ± 1	52 ± 4	57 ± 2	76 ± 4	45 ± 1	72 ± 16	46 ± 19
	−8°	42 ± 3	26 ± 1	52 ± 4	56 ± 2	76 ± 4	46 ± 1	72 ± 16	47 ± 19
	−9°	42 ± 3	21 ± 1	52 ± 4	55 ± 2	76 ± 4	47 ± 1	72 ± 16	48 ± 19
	−10°	42 ± 3	26 ± 1	52 ± 4	54 ± 2	76 ± 4	48 ± 1	72 ± 16	49 ± 19
+5°	0°	42 ± 6	36 ± 2	52 ± 3	64 ± 1	76 ± 1	38 ± 1	57 ± 14	43 ± 40
	+5°	42 ± 6	41 ± 2	52 ± 3	69 ± 1	76 ± 1	33 ± 1	57 ± 14	38 ± 40
	+10°	42 ± 6	46 ± 2	52 ± 3	74 ± 1	76 ± 1	28 ± 1	57 ± 14	33 ± 40
	−5°	42 ± 6	31 ± 2	52 ± 3	59 ± 1	76 ± 1	43 ± 1	57 ± 14	48 ± 40
	−10°	42 ± 6	26 ± 2	52 ± 3	54 ± 1	76 ± 1	48 ± 1	57 ± 14	53 ± 40
+10°	0°	42 ± 7	35 ± 6	52 ± 2	64 ± 1	76 ± 1	38 ± 1	45 ± 8	39 ± 31
	+5°	42 ± 7	40 ± 6	52 ± 2	69 ± 1	76 ± 1	33 ± 1	45 ± 8	44 ± 31
	+10°	42 ± 7	45 ± 6	52 ± 2	74 ± 1	76 ± 1	28 ± 1	45 ± 8	49 ± 31
	−5°	42 ± 7	30 ± 6	52 ± 2	59 ± 1	76 ± 1	43 ± 1	45 ± 8	34 ± 31
	−10°	42 ± 7	25 ± 6	52 ± 2	54 ± 1	76 ± 1	48 ± 1	45 ± 8	29 ± 31
−5°	0°	42 ± 4	36 ± 1	52 ± 7	64 ± 1	76 ± 1	38 ± 1	76 ± 14	38 ± 13

	+5°	42 ± 4	41 ± 1	52 ± 7	69 ± 1	76 ± 1	33 ± 1	76 ± 14	23 ± 13
	+10°	42 ± 4	46 ± 1	52 ± 7	74 ± 1	76 ± 1	28 ± 1	76 ± 14	28 ± 13
	−5°	42 ± 4	31 ± 1	52 ± 7	59 ± 1	76 ± 1	43 ± 1	76 ± 14	43 ± 13
	−10°	42 ± 4	26 ± 1	52 ± 7	54 ± 1	76 ± 1	48 ± 1	76 ± 14	48 ± 13
−10°	0°	42 ± 3	36 ± 1	52 ± 17	65 ± 9	76 ± 1	38 ± 1	76 ± 11	38 ± 10
	+5°	42 ± 3	41 ± 1	52 ± 17	70 ± 9	76 ± 1	33 ± 1	76 ± 11	33 ± 10
	+10°	42 ± 3	46 ± 1	52 ± 17	75 ± 9	76 ± 1	28 ± 1	76 ± 11	28 ± 10
	−5°	42 ± 3	31 ± 1	52 ± 17	60 ± 9	76 ± 1	43 ± 1	76 ± 11	43 ± 10
	−10°	42 ± 3	26 ± 1	52 ± 17	55 ± 9	76 ± 1	48 ± 1	76 ± 11	48 ± 10

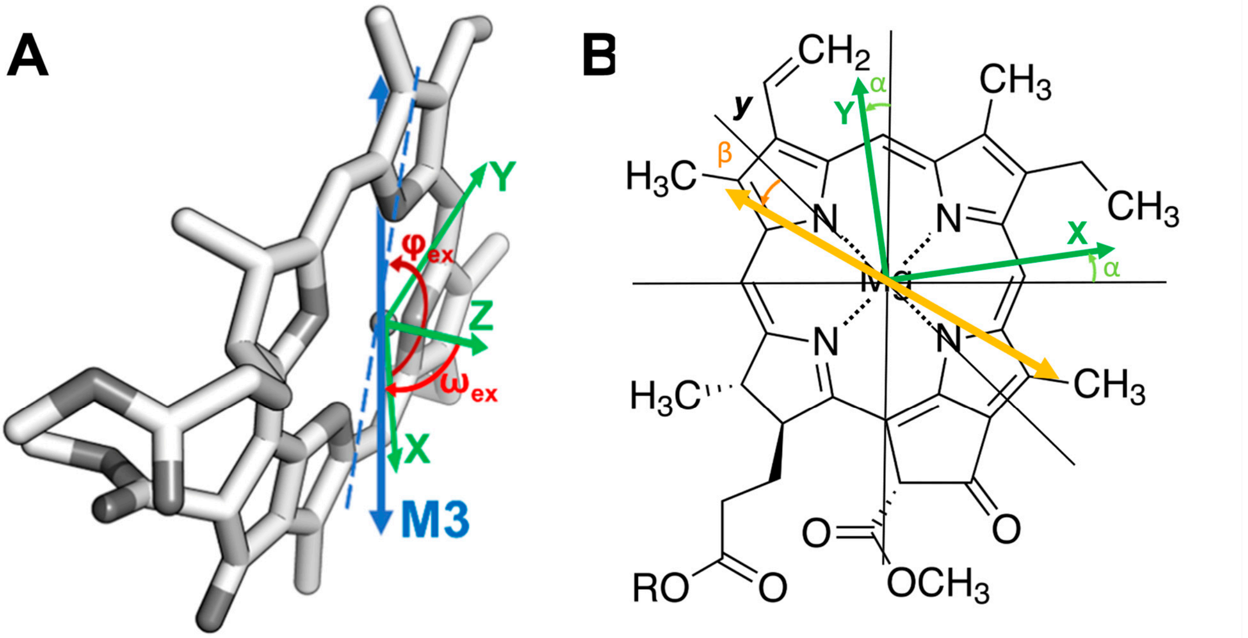

Figure S5. (**A**) Orientation of the ZFS axes system (green) relative to the M3 transition dipole moment (blue) in one monomer of Chl *a* in WSCP; the projection of the M3 moment on the molecular plane is reported as a dashed line, ω_{ex} and φ_{ex} angles (red and dark red, respectively) are shown. (**B**) Relative orientations of the ZFS axes (green), the molecular *y* axis (black), and the TDM axis of the Q_y transition (yellow). The molecular *z* axis points straight out of the molecular plane, towards the reader. The angles α and β from Table S2 are shown in light green and orange, respectively.

Nature of the second excitonic transition at 675 nm

Magnetophotoselection (MPS) results on WSCP obtained by photoexcitation at 675 nm were simulated considering the sum of two contributions arising from the M3 transition dipole moment (TDM) and a second TDM. Three TDM were considered as potential candidates for the second contribution:

A. The system can be described as a tetramer, and the contributing TDM is the one labelled as M2, with orientation (ω_{ex}=52°, ϕ_{ex}=72°) with respect to the ZFS axes.
B. The system can be described as a tetramer and the contributing TDM is the one labelled as M1, with orientation (ω_{ex}=42°, ϕ_{ex}=44°) with respect to the ZFS axes. This situation is considered since a rotation of Q_y in the chlorophyll plane can reverse the order of the oscillatory strengths of M1 and M2.
C. The system can be described as a dimer of non-interacting dimers, in which case only two excitonic transitions arise, one having the same orientation of M3 due to the symmetry of the system, labelled MH, and the second one with orientation (ω_{ex}=31°, ϕ_{ex}=78°) with respect to the ZFS axes, labelled ML. This description was

used to satisfactorily reproduce the absorption and circular dichroism spectra of WSCP [32].

Simulations of MPS spectra with complete polarization for the three possible TDM orientations are reported in Fig. S6. The simulations of the experimental data using both a contribution from M3 and either M1 or ML are reported in Fig. S7; in the simulations we keep the orientations (ω_{ex}, φ_{ex}) of the TDM relative to the ZFS axes fixed, while their relative weight is obtained through fitting (see Table S3).

The principal characteristics differentiating the TR-EPR spectra of WSCP collected by photoexcitation at the two wavelengths (658 nm *vs* 675 nm) are: 1) an enhanced Y transition in the spectrum obtained with light polarized parallel to the magnetic field at 675 nm, opposite to what can be observed at 658 nm (see Fig. 4C, main text); 2) the Z transition is more intense in the spectrum with light polarized perpendicular to the magnetic field at both wavelengths, but the difference between the two is smaller at 675 nm.

Only the fitting obtained with M2 and M3 satisfies both conditions above. Adding either M1 or ML fails to reproduce condition 1) above (see the asterisks in Figure S7) and meets condition 2) only at small weights of the second component (see Table S3).

Table S3. Simulation parameters used in the simulations reported in Figures S6. Angles defining the orientation of the ZFS axis system relative to the transition dipole moment (ω_{ex} and φ_{ex}); percentage of each spectral component (W). The other parameters that were kept constant for all species are: |D| = 30.9 mT; |E| = 3.65 mT; $p_{x/y/z}$ = 0.29/0.59/0.12; percentage of the polarized contribution, P = 57%.

	**ω_{ex} (°)**	**ϕ_{ex} (°)**	**W (%)**
M1	76	30	85
	42	44	15
ML	76	30	76
	31	78	24

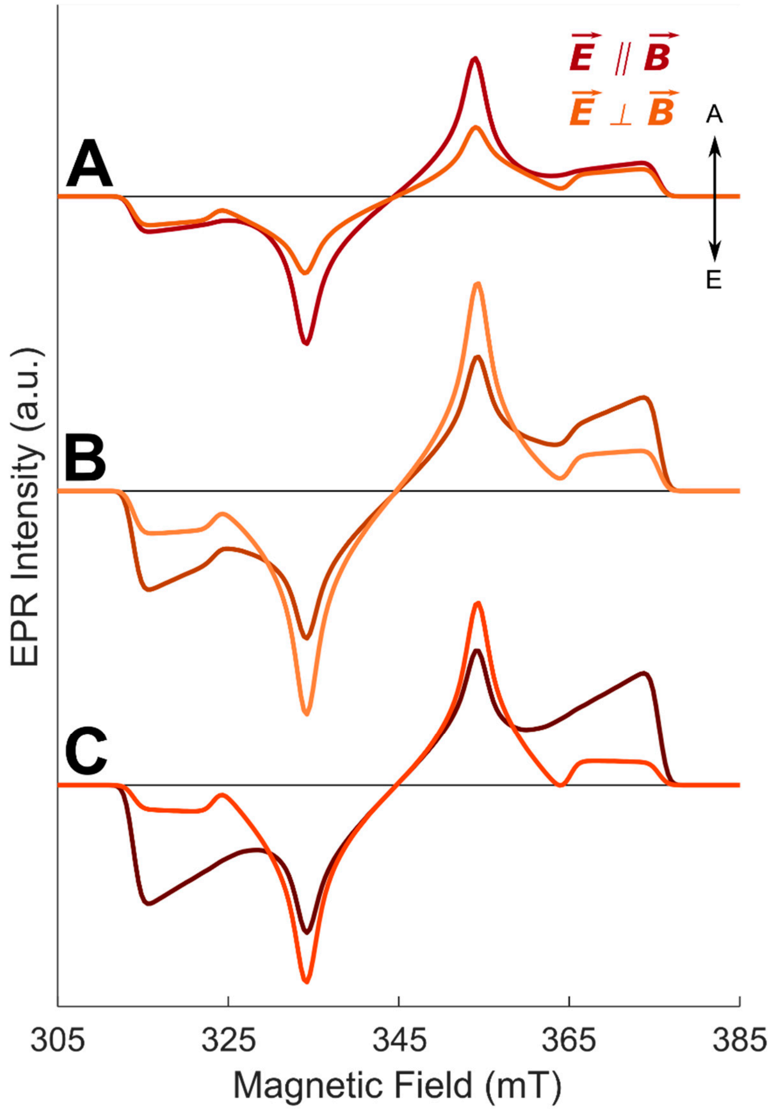

Figure S6. Simulation models of the magnetophotoselection TR-EPR spectra on WSCP obtained by photoexcitation of different excitonic transitions. Dark colours - laser polarized parallel to the magnetic field ($\vec{E} \parallel \vec{B}$); light colours – laser polarized perpendicular to the magnetic field ($\vec{E} \perp \vec{B}$); the arrows denote enhanced absorption (A) and emission (E). A Simulations of the triplet originating from the M2 excitonic transition in the coupled model. B Simulations of the triplet originating from the M1 excitonic transition in the coupled model. C Simulations of the triplet originating from the ML excitonic transition in dimer of non-interacting dimers model according to the hypothesis of Renger et al. [32]

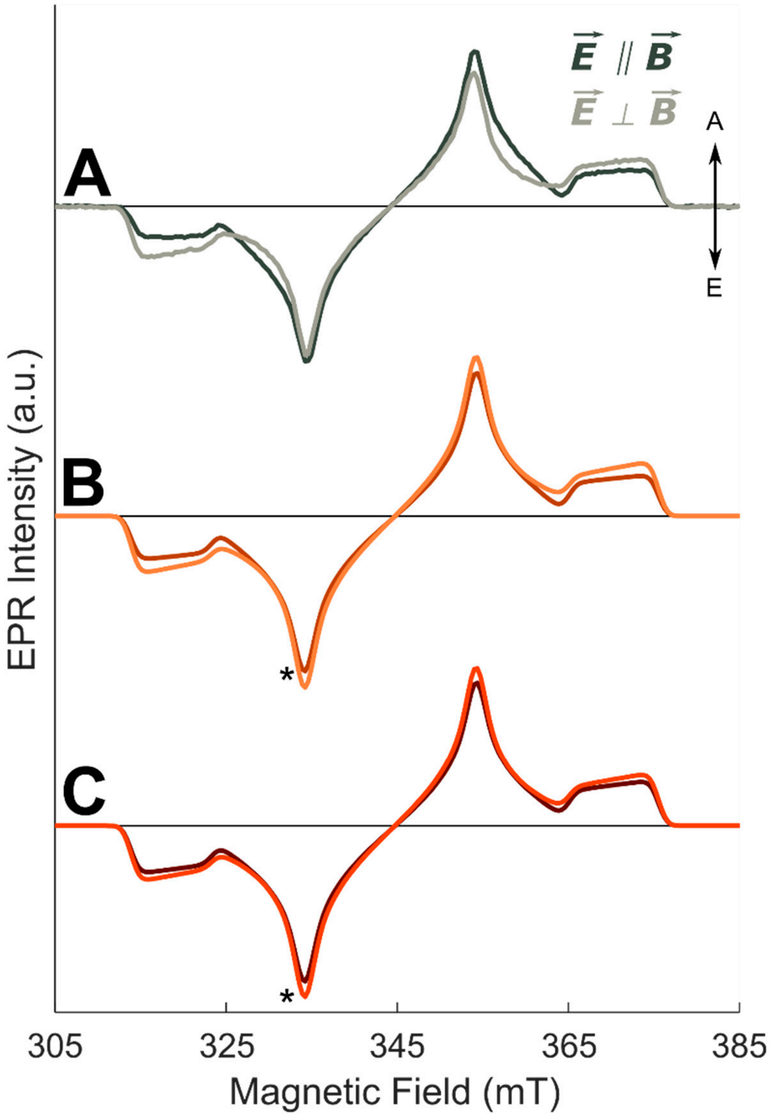

Figure S7. Simulation models of the magnetophotoselection TR-EPR spectra on WSCP obtained by photoexcitation at 675 nm. Dark colours - laser polarized parallel to the magnetic field ($\vec{E} \parallel \vec{B}$); light colours – laser polarized perpendicular to the magnetic field ($\vec{E} \perp \vec{B}$); the arrows denote enhanced absorption (A) and emission (E). **A** Experimental TR-EPR spectra. **B** Simulations considering a sum of triplets originating from the M3 and M1 excitonic transitions. **C** Simulations considering a sum of triplets originating from the MH and ML excitonic transitions in the dimer of non-interacting dimers model treated according to the hypothesis of Renger *et al.* [32]. The asterisks denote the critical point where the simulations fail to reproduce the trend observed for the experimental data.
